# Gut microbiota insights into human adaption to high‐plateau diet

**DOI:** 10.1002/imt2.6

**Published:** 2022-02-24

**Authors:** Yina Huang, Jinxin Liu, Hein Min Tun, Catherine Stanton, Tingtao Chen, Hani El‐Nezami, Hua Wei, Mingfu Wang, Qinglong Wu

**Affiliations:** ^1^ State Key Laboratory of Food Science & Technology Nanchang University Nanchang China; ^2^ College of Animal Science & Technology Nanjing Agricultural University Nanjing China; ^3^ School of Public Health The University of Hong Kong Hong Kong, SAR China; ^4^ APC Microbiome Ireland University College Cork Cork Ireland; ^5^ Institute of Translational Medicine Nanchang University Nanchang China; ^6^ School of Biological Sciences The University of Hong Kong Hong Kong, SAR China; ^7^ Institute of Public Health and Clinical Nutrition University of Eastern Finland Kuopio Finland; ^8^ Institute for Advanced Study Shenzhen University Shenzhen China; ^9^ Department of Pathology and Immunology Baylor College of Medicine Houston Texas USA; ^10^ Texas Children's Microbiome Center Texas Children's Hospital Houston Texas USA

## Abstract

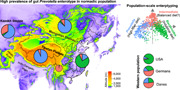

Modern genome analyses have identified unique genetic variations of indigenous people in terms of human adaptation to the low‐oxygen condition in high plateaus [1]. On the other hand, other key aspects such as diet have been an important consideration for human adaptation to such high‐plateau environments [2]. In ancient times, indigenous nomads often lack vegetables and fruits because of geographic limitations causing difficulties in trading agricultural products with low‐altitude people, for example, Tea‐Horse Road was an important but time‐consuming trade path in the past for Tibetans to obtain the dark tea from low‐altitude tea manufacturers. Through long‐term dietary self‐selection, dark tea becomes an essential part of nomadic diets in high plateaus and low‐altitude steppe areas (Figure 1A). Of note, consumption of fermented milk products is a tradition for thousands of years in nomadic diets (Figure 1A). Both tea and fermented milk are widely recognized to contain functional ingredients to confer health‐promoting benefits to humans, but further confirmation in nomadic populations is merited.

**Figure 1:**
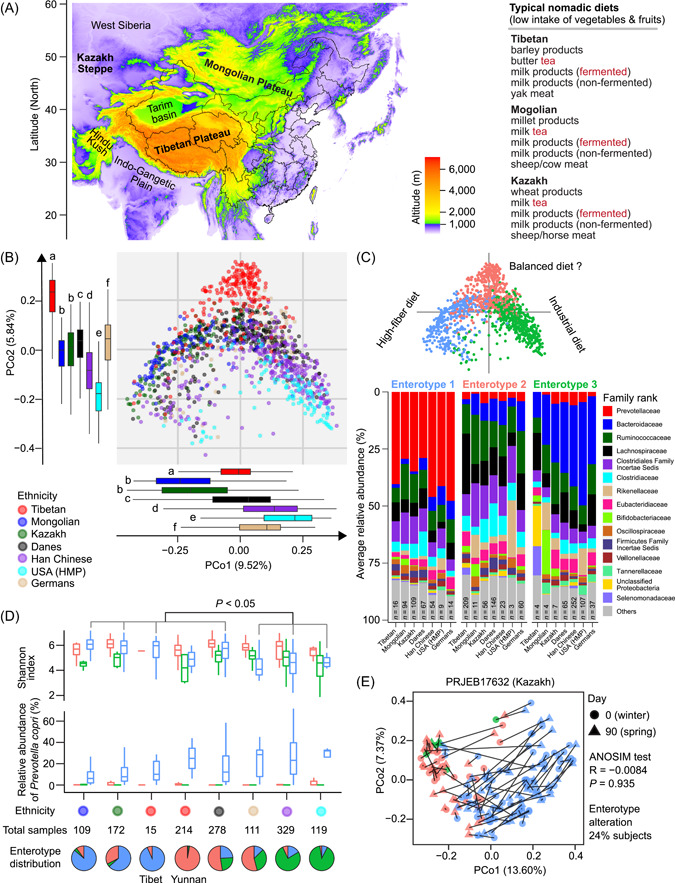
Gut *Prevotella* enterotype of high microbial diversity in people of nomadic regions. (A) Geographic location and dietary patterns of major nomadic regions. (B) High‐plateau people have a distinct gut microbiota profile compared to low‐altitude major ethnic groups. Beta‐diversity analysis was performed with Bray–Curtis dissimilarity profile for mOTUs‐based profiles for over 1000 publicly available shotgun metagenomes (NCBI Sequence Read Archive database), including PRJEB4336 (healthy Danes, *n* = 278), healthy Han Chinese (PRJEB6337, *n* = 145; PRJNA422434, *n* = 172), PRJEB17632 (Kazakh, *n* = 172; healthy Germans, *n* = 111), PRJNA48479 (HMP, *n* = 119), PRJNA328899 (Mongolian, *n* = 109), and Tibetan (PRJNA543906—Tibet, *n* = 27; PRJNA588513—Yunnan, *n* = 214). Lowercase (a)–(f) in the boxplot with no letters in common indicates a significant difference (*p* < 0.05; pairwise Wilcoxon test with Benjamini–Hochberg adjustment) between any two groups. (C) Dirichlet multinomial mixtures (DMM)‐based enterotyping of human metagenomic profiles (mOTUs genus‐level abundance profile) identifies three distinct gut enterotypes. (D) Microbial diversity (Shannon index), *Prevotella copri* abundance and enterotype distribution of each ethnic group. Pairwise Wilcoxon test was performed for comparing any two groups indicated in the plot. (E) Gut *Prevotella* enterotype is relatively stable in one Kazakh data set during seasonal shift. ANOSIM test using Bray–Curtis distance profile indicates no significant microbiome shift during longitudinal measurements. ANOSIM, analysis of similarities; mOTUs, marker gene‐based operational taxonomic units; NCBI, National Center for Biotechnology Information

Even after the advancement of transportation and food globalization, modern high‐altitude minorities usually maintain their nomadic dietary habits. From the standpoint of modern nutrition, nomadic diet is a minimally processed diet with low contents of vegetables and fruits and should not be regarded as a balanced diet, but it is quite distinct from the Western diet, a modern diet that contains high amounts of processed and prepackaged foods, red meat, and high‐fat and high‐sugar foods. Prior efforts have demonstrated the importance of host–diet–gut microbiota interactions in human health for a low‐altitude population with the Western diet. Thus, there is a need to dissect the interaction between nomadic diet and gut microbiota of high‐plateau minorities, and gut microbiota research might shed new light on human adaption to the high‐plateau environment linking to nomadic diet.

We performed a meta‐analysis of more than 1000 previously published shotgun metagenomes of adult people from nomadic regions of Tibetan and Mongolian Plateaus and Kazakh Steppe, and low‐altitude people from Europe, the United States, and China. To minimize the batch effect across different data sets, we did not perform assembly‐based metagenomic analysis that requires high sequencing depths, rather we applied marker gene‐based operational taxonomic units (mOTUs; version 2.6) that use 10 universal single‐copy marker genes for taxonomic profiling with high precision and low error [3]. Compared to the low‐altitude population, people from nomadic regions have distinct gut microbiota composition (Figure 1B), which is largely dominated by *Prevotella* (Figure 1C). While it is well known that industrial diet is responsible for the formation of *Bacteroides* enterotype in people receiving Western diet, it is surprising that many people commonly harbor *Prevotella* enterotype independent of nomadic regions (Figure 1C,D), which could be induced by high intake of cereal products in their diets [4]. More interestingly, enterotype analysis indicated that Tibetans from Yunnan (PRJNA588513) having a higher intake of diverse vegetables and fruits are different from Tibetans from Tibet (PRJNA543906) with traditional nomadic diet (Figure 1D). This suggests that dietary alteration could shift gut enterotype significantly. There have been several important studies demonstrating *Prevotella* is associated with exacerbation of autoimmune diseases, such as rheumatoid arthritis [5, 6, 7, 8]. It is highly speculative that the high prevalence of rheumatoid arthritis [9, 10] in the Tibetan population could be associated with their common gut *Prevotella* enterotype, thus further studies are required to validate such causation and to investigate whether the dietary intervention could be a potential strategy to effectively prevent the occurrence of rheumatoid arthritis. Of note, tea [11] and fermented milk [12] have been shown to lower host systematic inflammation though they could have limited alterations to gut microbiota composition. It is quite interesting to see the frequent consumption of tea and fermented milk in Tibetan, Mongolian, and Kazakh populations (Figure 1A), but linking such dietary habits to reduced inflammation is questionable and requires a systematic design of diet intervention to confirm it. Interestingly, we observed a significantly (*p* < 0.05; Wilcoxon test) higher alpha‐diversity in people with *Prevotella* enterotype in nomadic regions than that in low‐altitude people of the same enterotype (Figure 1D). While it is not clear whether such higher microbial diversity could be helpful against *Prevotella*‐associated inflammation, it is likely to play a role in host health since the loss of microbial diversity is associated with several human conditions [13]. In addition, as shown in Figure 1E, we found that gut enterotype was largely unaltered during the seasonal shift in one Kazakh data set (PRJEB17632) because nomadic dietary habits could be commonly excised by modern Kazakh. This is a quite different observation from a prior report on seasonal variations in gut microbiota composition in the Hadza hunter–gatherers of Tanzania [14]. Taken together, a distinct gut *Prevotella* enterotype of high microbial diversity is highly prevalent in people from nomadic regions.

Our preliminary evidence from population‐scale gut microbiota meta‐analysis is encouraging, but future research efforts are necessary to understand human adaptation to nomadic diet with insights into host–diet–gut microbiota interactions. Most likely, long‐term high cereal consumption could explain the dominance of *Prevotella* enterotype in people of nomadic regions where carbohydrate‐based diet is more affordable in addition to its sufficient energy and biofunctionalities [4, 15]. For example, highland barley is rich in beta‐glucan and its high molecular weight form has been clinically shown to reduce blood cholesterol levels via the enhanced bile acids synthesis in the liver and the increased fecal excretion of secondary bile acids [16]. Low levels of blood cholesterol inhibit the formation of plaques in the blood vessels, which indirectly favors the control of blood pressure that is the top risk factor driving the most death and disability in nomadic regions, thus the high intake of barely products in the Tibetan population seems to be beneficial for host health. However, whether a *Prevotella* enterotype of high microbial diversity is responsible for the increased biotransformation of secondary bile acids is of great interest but remains largely unknown. Additionally, a high‐fat diet has been shown to reduce *Prevotella* abundance [15, 17]; we noticed this conflicting observation typically in *Prevotella*‐dominated Tibetan because they consume Yak butter tea and barely‐based food on a daily basis. Butter tea is an emulsified beverage through physical processing, so it is not clear whether fat absorption is prohibited by emulsification or by tea ingredients, that is, tea polyphenols and their derivatives. Importantly, *Prevotella* enterotype is associated with the host genotype of rheumatoid arthritis before disease onset [6, 7], enhances disease susceptibility, and exacerbates disease severity of rheumatoid arthritis and colonic inflammation [5, 8]. Because rheumatoid arthritis is the second highly prevalent (8%) chronic disease in Tibet [10], it becomes important to understand how the host responds to proinflammatory *Prevotella* enterotype in the gut and what components in nomadic diet could reverse such inflammation status. For example, tea ingredients have been documented as anti‐inflammatory agents, whereas a recent human dietary intervention study for low‐altitude individuals of *Bacteroides* enterotype reported that consumption of fermented foods increased gut microbial diversity and decreased inflammatory markers [12], but whether such health‐promoting benefits could be recapitulated in individuals of *Prevotella* enterotype remains to be elucidated. Overall, nomadic diet is likely associated with *Prevotella* expansion and is associated with several major chronic diseases in people of nomadic regions but requires further systematic assessment on host–diet–gut microbiota interactions.

It is necessary to understand the pros and cons of traditional nomadic diets in terms of host health before establishing new nutritional guidelines for people of nomadic regions. Despite technical and analytic advancements of gut microbiota research for the past decade, there have been several challenges in conducting gut microbiota research for high‐plateau populations. Thus, we highlight those challenges and discuss corresponding strategies to tackle them.

First, infrastructure advancement and food globalization have significant impacts on the accessibility of food resources, including fresh vegetables and fruits, which ultimately change gut microbiota composition as evidenced by two Tibetan data sets (Yunnan and Tibet; Figure 1D) that we included in our meta‐analysis. At the current stage, higher food accessibility shifts their dietary pattern thus limiting the eligibility and the size of subjects maintaining traditional nomadic diets to be recruited for gut microbiota research, especially if disease prevalence and pathogenesis are the major focus of host–diet–gut microbiota association study. To avoid the effects of nonnomadic foods, longitudinal, randomized cross‐over diet intervention with nomadic and nonnomadic foods could be considered, but the duration of each intervention should be carefully considered. If necessary, a cross‐over trial could be repeated immediately after the first trial to gain more insights and might be useful to assess the stability of gut microbiota and the effect of seasonal cycling.

Second, the size of nomadic regions, including Tibetan and Mongolian Plateaus and Kazakh Steppe, is over 10 million square meters, and traditional nomads remain unsettled making gut microbiota research extremely difficult; that is, clinical visits, follow‐up, and longitudinal sampling are of major concerns. Appropriate stool shipping is another concern because preserving microbial composition and bioactivity is important for further DNA/RNA extraction, sequencing, and microbial isolation. A virtual clinical visit could be highly helpful because telecommunications are now largely covered for high plateaus, thus this approach seems to be useful for people with a nomadic lifestyle. To tackle the sampling challenge, there could be two potential solutions to include stool collection during: (1) large disease screenings. For example, screening of hydatidosis, a major zoonosis of parasitic infection in Tibet, has been performed for more than three million Tibetan people from 2016 to 2017 and (2) national health service surveys. Those surveys of high administrative level are held regularly and are suitable for such purpose, but require study permission and detailed administrative coordination. Those governmental mission‐orientated screenings and surveys are highly recommended because additional clinical metadata could be collected for disease‐microbiota associations with consideration of geographic influence, human development (particularly children and elderly), gender difference, dietary information, and so forth. For example, the prevalence of rheumatoid arthritis is more common in the elderly compared with the younger population in Tibet, thus requiring an age‐stratified analysis.

Finally, gut microbiota profiling and bacterial isolation are big challenges. Because targeted (16S ribosomal RNA [rRNA] and Internal Transcribed Spacer [ITS]) metagenomic analysis is not appropriate for precision compositional and functional profiling, a shotgun metagenomic analysis should be considered for such profiling. However, there is a large proportion of uncultured bacteria, including Clostridiales Family Incertae Sedis and Firmicutes Family Incertae Sedis, in the high‐plateau population. Since gut microbiota composition of the high‐plateau population is very unique compared to low‐altitude people, the computational effort is required to construct MAGs from shotgun metagenomes of the high‐plateau population. However, MAGs are consensus sequences constructed from the phylogenetic closely related strains or species of high identity and the genome completion of MAGs is usually not comparable to single genomes of cultivable microbes thus limiting further strain‐level and genome‐scale characterization. Future studies also need to focus on the isolation of gut commensal microbes from high‐plateau people, and preserving live gut anaerobic microbes during sample collection and shipping is essential for such a task. Since metabolic reconstruction for MAGs could potentially predict the essential nutrients for microbial growth, the computational effort might help the design of a culture medium for targeted isolation.

Taken together, high‐plateau people have an identical gut microbiota of *Prevotella* enterotype, and such proinflammatory enterotype is generally thought to be associated with nomadic diet patterns. Several characteristic food ingredients, including tea and fermented foods, in nomadic diet, have been shown to be helpful to tackle *Prevotella*‐centered chronic diseases but require a further comprehensive assessment of host–diet–gut microbiota interactions. However, gut microbiota research for people of high‐plateau regions needs to consider several unneglectable challenges, and such effort is to advance current nutritional guidelines and dietary interventions for promoting their health status.

## CONFLICT OF INTERESTS

The authors declare that there are no conflict of interests.

## AUTHOR CONTRIBUTIONS

Qinglong Wu and Mingfu Wang conceived the idea. Yina Huang, Jinxin Liu, and Qinglong Wu collected data and performed analysis. Hein Min Tun, Catherine Stanton, Tingtao Chen, Hani El‐Nezami, and Hua Wei interpreted and discussed results. All authors contributed to the writing of the manuscript.

## Data Availability

Shotgun metagenomic data used in this meta‐analysis are available from the NCBI Sequence Read Archive database with accession numbers indicated in this paper. The figures related tables and scripts were deposited in https://github.com/qinglong89/Nomad-Prevotella.
